# Combination of Radiosensitivity Gene Signature and PD-L1 Status Predicts Clinical Outcome of Patients With Locally Advanced Head and Neck Squamous Cell Carcinoma: A Study Based on The Cancer Genome Atlas Dataset

**DOI:** 10.3389/fmolb.2021.775562

**Published:** 2021-12-14

**Authors:** Dongjun Dai, Yinglu Guo, Yongjie Shui, Jinfan Li, Biao Jiang, Qichun Wei

**Affiliations:** ^1^ Department of Radiation Oncology, The Second Affiliated Hospital, Zhejiang University School of Medicine, Hangzhou, China; ^2^ Department of Pathology, The Second Affiliated Hospital, Zhejiang University School of Medicine, Hangzhou, China; ^3^ Department of Radiology, Second Affiliated Hospital, School of Medicine, Zhejiang University, Hangzhou, China

**Keywords:** locally advanced head and neck squamous cell carcinoma, The Cancer Genome Atlas, radiosensitivity, PD-L1, B cells, histone deacetylase inhibitor

## Abstract

**Aim:** The aim of our study was to investigate the potential predictive value of the combination of radiosensitivity gene signature and PD-L1 expression for the prognosis of locally advanced head and neck squamous cell carcinoma (HNSCC).

**Methods:** The cohort was selected from The Cancer Genome Atlas (TCGA) and classified into the radiosensitive (RS) group and radioresistant (RR) group by a radiosensitivity-related gene signature. The cohort was also grouped as PD-L1-high or PD-L1-low based on PD-L1 mRNA expression. The least absolute shrinkage and selection operator (lasso)-based Cox model was used to select hub survival genes. An independent validation cohort was obtained from the Gene Expression Omnibus (GEO) database.

**Results:** We selected 288 locally advanced HNSCC patients from TCGA. The Kaplan–Meier method found that the RR and PD-L1-high group had a worse survival than others (*p* = 0.033). The differentially expressed gene (DEG) analysis identified 553 upregulated genes and 486 downregulated genes (*p* < 0.05, fold change >2) between the RR and PD-L1-high group and others. The univariate Cox analysis of each DEG and subsequent lasso-based Cox model revealed five hub survival genes (*POU4F1*, *IL34*, *HLF*, *CBS*, and *RNF165*). A further hub survival gene-based risk score model was constructed, which was validated by an external cohort. We observed that a higher risk score predicted a worse prognosis (*p* = 0.0013). The area under the receiver operating characteristic curve (AUC) plots showed that this risk score model had good prediction value (1-year AUC = 0.684, 2-year AUC = 0.702, and 3-year AUC = 0.688). Five different deconvolution methods all showed that the B cells were lower in the RR and PD-L1-high group (*p* < 0.05). Finally, connectivity mapping analysis showed that the histone deacetylase (HDAC) inhibitor trichostatin A might have the potential to reverse the phenotype of RR and PD-L1-high in locally advanced HNSCC (*p* < 0.05, false discovery rate <0.1).

**Conclusion:** The combination of 31-gene signature and the PD-L1 mRNA expression had a potential predictive value for the prognosis of locally advanced HNSCC who had RT. The B cells were lower in the RR and PD-L1-high group. The identified risk gene signature of locally advanced HNSCC and the potential therapeutic drug trichostatin A for the RR and PD-L1-high group are worth being further studied in a prospective homogenous cohort.

## Introduction

Head and neck cancer accounts for more than 650,000 cases and 330,000 deaths worldwide (Bray et al., 2020). Ninety percent of head and neck cancer is head and neck squamous cell carcinoma (HNSCC). HNSCC consists of a series of squamous cell cancers that developed from the mucosal surfaces of the upper aerodigestive tract, including the oral cavity, pharynx, larynx, and sinonasal tract. Nearly 60% of patients of HNSCC were diagnosed with locally advanced stage (III and IVA-B) (Vokes et al., 1993).

Radiotherapy (RT) is widely used as adjuvant therapy for locally advanced HNSCC in addition to surgery. However, the 5-year overall survival of locally advanced HNSCC remains poor, as a high local recurrent rate (50%) occurs during the disease process (Lyu et al., 2019). Recently, immunotherapy is widely considered an important strategy for the treatment of later-stage cancers. Clinical trials have demonstrated that immunotherapy improves overall survival and progression-free survival in metastatic and locally advanced cancers (Pennock and Chow, 2015). Immune checkpoint inhibitors (ICIs) pembrolizumab (MK-3475) and nivolumab (BMS-936558), which target the programmed death 1 (PD1)/programmed death ligand 1 (PD-L1) pathway, were approved for HNSCC by the US Food and Drug Administration (FDA). These two drugs were also introduced by the National Comprehensive Cancer Network as standard therapeutic methods for HNSCC (Ran and Yang, 2017). However, the response rate of ICIs in cancers is only 20%–30% (Topalian et al., 2012). Interestingly, the combination of RT therapy and immunotherapy had a synergistic effect on the treatment of cancers (Chajon et al., 2017). RT could induce the damage of cancer cells and promote tumor-specific antigens, which make the tumor visible to immune surveillance and enhance the priming and activation of cytotoxic T cells (Wang et al., 2018).

The major ICIs are the antibodies for PD-1/PD-L1 signaling pathway. The PD-1 is expressed on the surface of immune-related lymphocytes and functions as a T-cell checkpoint. It binds to PD-L1 that is often expressed on tumor cells, which subsequently inhibits the host immune response (Garber et al., 2016). The expression of PD-L1 was observed to negatively affect the prognosis of cancer patients (Thompson et al., 2004; Ghebeh et al., 2006; Xiang et al., 2018). The high PD-L1 expression was also identified to be associated with radiosensitivity (Lyu et al., 2019).

With the development of genome sequencing technology, several radiosensitivity-related gene signatures were identified (Kim et al., 2012; Ghashghaei et al., 2019; You et al., 2019), which were useful to stratify the radioresistant (RR) and radiosensitive (RS) patients. The current study was aimed to combine the radiosensitivity-related gene signatures and the expression of PD-L1 status to classify different groups of locally advanced HNSCC and then to compare the clinical outcomes, genome profile, and immune cell infiltration in these groups. We also constructed RR- and PD-L1-high-related risk gene signatures to predict the prognosis of locally advanced HNSCC, along with the identification of possible effective drugs.

## Materials and Methods

### Obtaining Locally Advanced Head and Neck Squamous Cell Carcinoma Data and the Clustering

The Cancer Genome Atlas (TCGA) HNSCC (TCGA dataset for HNSCC) RNA-seq data were acquired from the Xena database (Mary Goldman et al., 2019). Only HNSCC patients with both RNA count data and clinical information were included. The locally advanced HNSCC patients were defined as stage III, IVA, and IVB patients. The RNA count data were normalized by “TMM” method (McCarthy et al., 2012) and transformed by “voom” method from “limma” R package (Ritchie et al., 2015). The prognostic validation cohort was obtained from the Gene Expression Omnibus (GEO) database (GSE65858), which included HNSCC microarray data and clinical information.

A radiosensitivity-related 31-gene signature, which was identified based on a meta-analysis that included four different microarrays using NCI-60 cancer cell lines (Kim et al., 2012), was used to perform the clustering process. This RS signature was validated among patients with breast cancer (Jang and Kim, 2017), lower-grade glioma (Jang and Kim, 2018), and glioblastoma (Jang and Kim, 2020). The 31 genes of this signature included *ACTN1*, *ANXA2*, *ANXA5*, *ARHGDIB*, *CAPNS1*, *CBR1*, *CCND1*, *CD63*, *COR O 1A*, *CXCR4*, *DAG1*, *EMP2*, *HCLS1*, *HTRA1*, *ITGB5*, *LAPTM5*, *LRMP*, *MYB*, *PFN2*, *PIR*, *PKM*, *PTMS*, *PTPRC*, *PTPRCAP*, *PYGB*, *RAB13*, *RALB*, *SCRN1*, *SQSTM1*, *TWF1*, and *WAS*. The clustering process was performed by “kmeans” method, and two clusters were obtained. The Kaplan–Meier (KM) method was applied to the clusters, with a log-rank *p*-value calculated. The cluster with a worse overall survival was defined as RR, and the cluster with a better overall survival was defined as RS. The KM plots were also drawn for locally advanced HNSCC patients with or without RT.

### Grouping of Radiosensitivity-Related Clusters With PD-L1 Expression

The optimal cutoff was explored for the PD-L1 (CD274) expression of overall locally advanced HNSCC patients when the KM methods were applied. The high and low groups of PD-L1 were then defined based on the best cutoff value. The combination of radiosensitivity-related clusters with PD-L1 expression revealed the following groups: RR and PD-L1-high group, RR and PD-L1-low group, RS and PD-L1-high group, and RS and PD-L1-low group. The KM plots of these combined groups were drawn for overall locally advanced HNSCC patients and patients with or without RT.

To further explore the detailed molecular profiles of the RR and PD-L1-high group, which had a worse overall survival than other groups, the groups were classified into the RR and PD-L1-high group and the others.

### Multivariate Cox Analysis for Locally Advanced Head and Neck Squamous Cell Carcinoma

A multivariate Cox analysis for the overall, RT, and non-RT groups of locally advanced HNSCC patients was performed. The radiosensitivity, PD-L1 expression, and clinical variables that comprised age, gender, grade, stage, T and N stage, HPV and P16 status, grade, and RT experience were included in the multivariate Cox analysis.

### Identification of Differentially Expressed Genes Between the Radioresistant and PD-L1-High Group and the Other Groups

The “limma” R package was used to identify the differentially expressed genes (DEGs) between the RR and PD-L1 group and the others. The DEGs were defined as the genes with a *p*-value of less than 0.05 and a fold change of over 2. The volcano plot was drawn to visualize the results of DEG identification. The DEGs were further used for the next functional analysis and survival analysis.

### Functional Analysis of Differentially Expressed Genes

The functional analyses of DEGs were performed by gene ontology (GO) analysis and Kyoto Encyclopedia of Genes and Genomes (KEGG) analysis. The GO analysis consists of items that belong to biological processes (BPs), cellular components (CCs), and molecular functions (MFs). *p*-Value <0.05 and q-value <0.05 were set as the cutoff values. Besides, the results of logFC from the DEG analysis were used for Gene Set Enrichment Analysis (GSEA). *p*-Value <0.05 was set as the cutoff for GSEA.

### Survival Analysis of Each Differentially Expressed Gene and Hub Survival Gene Selection

The univariate Cox analysis was applied for each DEG for locally advanced HNSCC patients who had RT. For each univariate Cox analysis, the patients were divided into high and low groups based on the median expression value of the DEG. *p*-Value <0.05 was considered statistically significant. The univariate Cox analysis was also applied to the locally advanced HNSCC patients from the GEO database.

The intersect survival-related DEGs from TCGA and GEO databases were then analyzed by a least absolute shrinkage and selection operator (lasso) analysis to select hub survival genes (10-fold cross-validation was used).

### Risk Score Model Construction and Validation

A risk score model was built based on hub survival genes by using the following formula: (*βi* × Exp*i*) (*i* = the number of hub survival-related genes). The KM method together with the identification of the optimal cutoff value was applied for survival analysis of the risk score for locally advanced HNSCC patients who had RT. The area under the receiver operating characteristic curve (AUC) plots were drawn to estimate the reliability of the risk score. The risk score model was validated by the GEO database.

### Estimation of Immune Cell Fractions

The Fragments Per Kilobase Million (FPKM) data of the locally advanced HNSCC were downloaded from Xena database and transformed to Transcripts Per Million (TPM) value. Deconvolution of cell-type fractions from RNA-seq data was calculated by xCell analysis (Aran et al., 2017), quantiseq (Finotello et al., 2019), TIMER (Li et al., 2020a), MCP-counter (Becht et al., 2016), and epic algorithms (Racle et al., 2017). The comparison was performed between the RR and PD-L1-high group and others.

### Potential Therapeutic Drug Identification for Radioresistant and PD-L1-High Group

CMap analysis is a method that uses a reference drug-specific gene expression profile dataset and compares it with a disease-specific gene expression profile to identify potential drugs for a specific phenotype (Lamb, 2007; Musa et al., 2018). In the current study, the “DrInsight” R package was used to perform the CMap analysis, which aimed to find potential drugs targeted for the RR and PD-L1-high group (Chan et al., 2019). The reference profile for CMap analysis is the widely used CMap dataset, which consists of cellular signatures that catalog transcriptional responses of human cells to chemical and genetic perturbation (Subramanian et al., 2017). The function “get.cmap.ref” was used to load the CMap data matrix. The function “drug.ident” was used to connect drugs (compounds) in the CMap dataset with query data (disease phenotype, RR and PD-L1-high group). The results of the *t*-test statistic scores from the “limma” analysis were used as input data in this analysis. The identified drug should have a *p*-value <0.05 and a false discovery rate (FDR) <0.1.

### Statistical Analysis

The R-4.0.2 was used for all the analyses. The chi-square analysis was applied for the clinical information of locally advanced HNSCC patients with or without RT. The KM method was performed by “survminer” R package. The optimal cutoff was defined by the “surv_cutpoint” function from the “survminer” R package. The lasso method was performed by “glmnet” R package. The deconvolution of cell-type fractions was calculated by “immunedeconv” R package. The functional analyses and GSEA were performed by “clusterProfiler” R package. The AUC plot was drawn by “ROCR” R package. The Wilcoxon analysis and Kruskal–Wallis analysis were used for the comparisons between two groups and three groups, respectively.

## Results

### Gene Signature Clustering and PD-L1 Grouping

**FIGURE 1:**
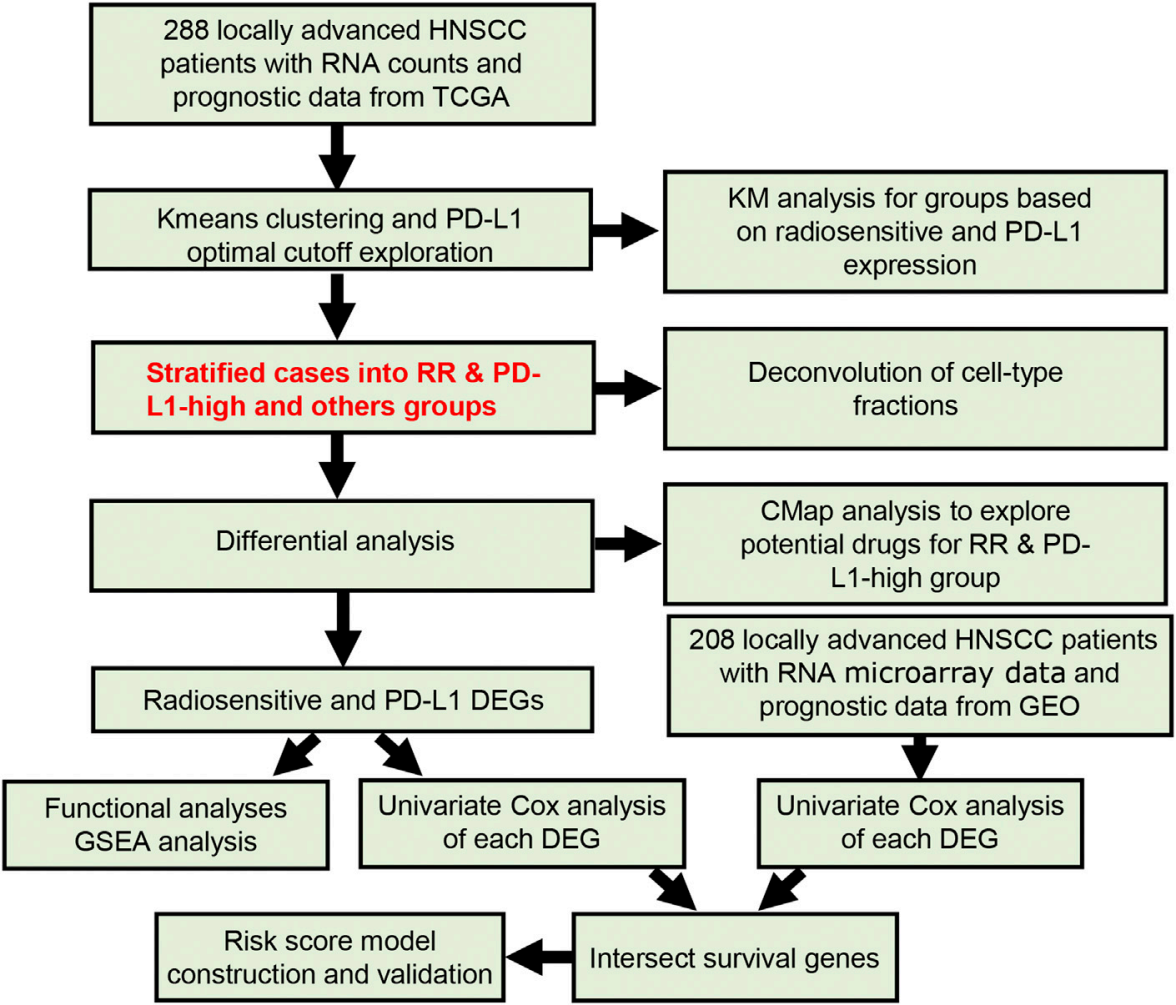
The flowchart of the current study.

**FIGURE 2:**
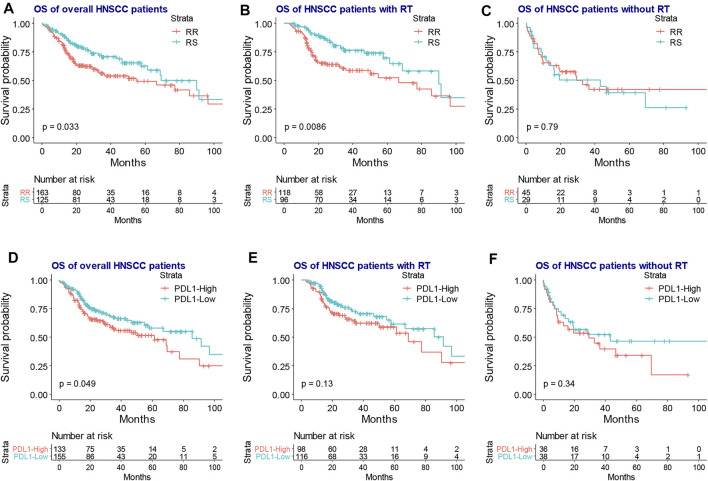
The KM plot of RR and RS clusters and the expression level of PD-L1 in the overall, RT, and non-RT groups of locally advanced HNSCC patients. The KM plot shows that the RR group had worse overall survival than the RS group in the overall **(A)** and RT groups **(B)** but not in the non-RT group **(C)**. The high expression PD-L1 was associated with inferior overall survival in the overall patients **(D)** but had no association with the prognosis of the RT group **(E)** and non-RT group **(F)**. KM, Kaplan–Meier; RR, radioresistant; RS, radiosensitive; RT, radiotherapy; HNSCC, head and neck squamous cell carcinoma; HR, hazard ratio.

**FIGURE 3:**
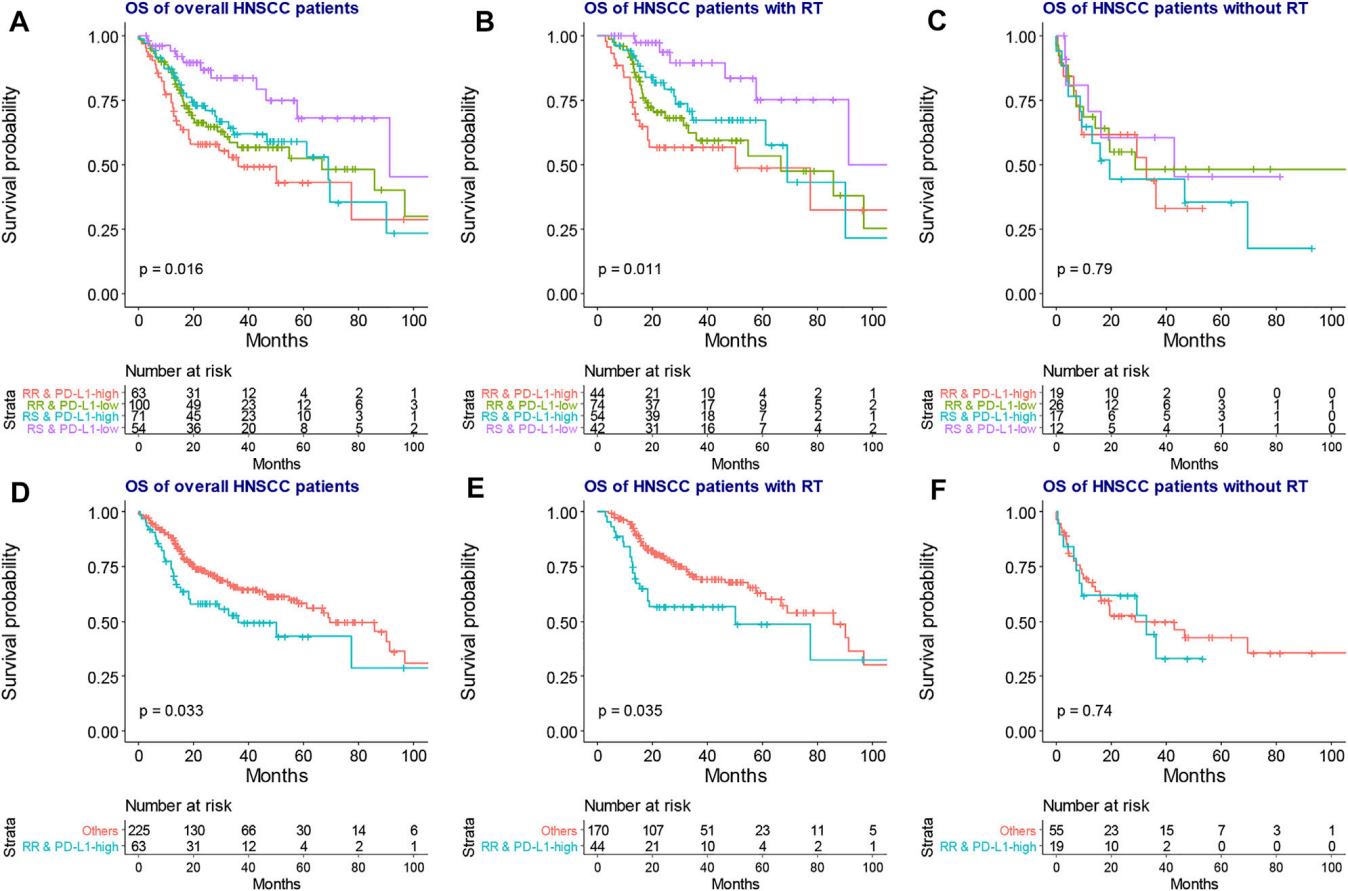
The KM plot of overall survival among four groups based on RT sensitivity and PD-L1 expression. The four groups exhibited significantly different overall survival in the overall **(A)** and RT groups **(B)** but not in the non-RT group **(C)**. The RR and PD-L1-high group had significantly worse overall survival than the “others” group in the overall **(D)** and RT groups **(F)** but not in the non-RT group **(G)**. KM, Kaplan–Meier; RT, radiotherapy.

As shown in Figure 1, RNA count data and clinical information of 288 locally advanced HNSCC patients were collected from TCGA database. The median age of locally advanced HNSCC patients was 61 years (interquartile range = 14). The median overall survival of locally advanced HNSCC patients was 22.65 months (interquartile range = 30.08). After the clustering analysis by 31 radiosensitivity-related genes, two clusters were identified. KM method found a significant prognostic difference between the two clusters (Figure 2A, *p* = 0.033). The same analysis was also applied for locally advanced HNSCC patients with or without RT. The significant prognostic difference was observed to be only existing in the RT group (Figure 2B, *p* = 0.0088) but not in the non-RT group (Figure 2C, *p* = 0.79). Higher expression of PD-L1 was also observed to be associated with worse overall survival in the overall group (Figure 2D, *p* = 0.049) but not in the RT or non-RT group (Figures 2E,F, *p* = 0.13 and 0.34 for the RT and non-RT groups, respectively), which might be a result of the inadequate patient sample. Based on the radiosensitivity analysis and the expression level of PD-L1 of locally advanced HNSCC patients, the overall patients were stratified into four groups, as follows: RR and PD-L1-high group, RR and PD-L1-low group, RS and PD-L1-high group, and RS and PD-L1-low group. Among these four groups, the KM method observed significant prognostic differences in overall patients (Figure 3A, *p* = 0.016) and patients with RT (Figure 3B, *p* = 0.011) but not in patients without RT (Figure 3C, *p* = 0.79). Further focus was on the survival of the RR and PD-L1-high group, which had the worst overall survival than others. It was observed that the RR and PD-L1-high group had worse survival than others in overall patients (Figure 3D, *p* = 0.033) and patients with RT (Figure 3E, *p* = 0.035) but not in patients without RT (Figure 3F, *p* = 0.74). Therefore, the following analyses, including the multivariate Cox analysis, DEG analysis, immune cell fraction identification, and target drug exploration, were applied in the comparisons between the RR and PD-L1-high group and others.

### Multivariate Cox Analysis for Locally Advanced Head and Neck Squamous Cell Carcinoma Patients

The baseline characteristics of locally advanced HNSCC patients are listed in Table 1. Compared with the non-RT group, the RT group had patients with younger age (RT vs. non-RT = 0.551 vs. 0.311, *p* < 0.001), more stage IVa and IVb (RT vs. non-RT = 0.757 vs. 0.595, *p* = 0.012), and more HPV negative status (RT vs. non-RT = 0.159 vs. 0.054, *p* = 0.024). No other significant results were found between the RT and non-RT groups (*p* > 0.05).

**TABLE 1:** The clinical characteristics of overall, RT, and non-RT groups of patients with locally advanced HNSCC.

Characteristics	Overall HNSCC	RT group	Non-RT group	*p*-Value
	No. of patients (%)	No. of patients (%)	No. of patients (%)	
Age				**<0.001**
High	147 (0.51)	96 (0.449)	51 (0.689)	
Low	141 (0.49)	118 (0.551)	23 (0.311)	
Gender				0.242
Female	62 (0.215)	42 (0.196)	20 (0.27)	
Male	226 (0.785)	172 (0.804)	54 (0.73)	
Stage				**0.012**
Stage III	82 (0.285)	52 (0.243)	30 (0.405)	
Stage IV	206 (0.715)	162 (0.757)	44 (0.595)	
T stage				0.305
T1–2	50 (0.174)	35 (0.164)	15 (0.203)	
T3	103 (0.358)	73 (0.341)	30 (0.405)	
T4	135 (0.469)	106 (0.495)	29 (0.392)	
N stage				0.203
N0	98 (0.34)	69 (0.322)	29 (0.392)	
N1	66 (0.229)	46 (0.215)	20 (0.27)	
N2–3	119 (0.413)	94 (0.439)	25 (0.338)	
NA	5 (0.017)	5 (0.023)	-	
HPV status				**0.024**
Negative	38 (0.132)	34 (0.159)	4 (0.054)	
Positive	11 (0.038)	10 (0.047)	1 (0.014)	
NA	239 (0.83)	170 (0.794)	69 (0.932)	
P16 status				0.056
NA	223 (0.774)	159 (0.743)	64 (0.865)	
Negative	48 (0.167)	39 (0.182)	9 (0.122)	
Positive	17 (0.059)	16 (0.075)	1 (0.014)	
Grade				0.051
G1–2	207 (0.719)	147 (0.687)	60 (0.811)	
G3–4	71 (0.247)	57 (0.266)	14 (0.189)	
NA	10 (0.035)	10 (0.047)		
Margin status				0.0634
NA	34 (0.118)	27 (0.126)	7 (0.095)	
Close	25 (0.087)	14 (0.065)	11 (0.149)	
Negative	199 (0.691)	147 (0.687)	52 (0.703)	
Positive	30 (0.104)	26 (0.121)	4 (0.054)	
Radiosensitivity and PD-L1 groups				0.451
Others	225 (0.781)	170 (0.794)	55 (0.743)	
RR and PD-L1-high	63 (0.219)	44 (0.206)	19 (0.257)	
Radiotherapy				
No	74 (0.257)	-	-	-
Yes	214 (0.743)	-	-	
PD-L1 level				
High	134 (0.465)	98 (0.458)	36 (0.486)	0.773
Low	154 (0.535)	116 (0.542)	38 (0.514)	

Note. The significant results of chi-square analysis are bolded. The *p*-value describes the difference between RT and non-RT groups.

RT, radiotherapy; HNSCC, head and neck squamous cell carcinoma; RR, radioresistant.

Multivariate Cox analyses were performed for overall locally advanced HNSCC and patients with or without RT. As shown in Table 2, for the overall group, male patients were found to have better survival than female patients (hazard ratio (HR) = 0.59, 95%CI = 0.38–0.92, *p* = 0.02), patients with positive margin status had worse overall survival than patients with negative margin status (HR = 2.73, 95%CI = 1.57–4.75, *p* < 0.001), the RS group had better survival than the RR group (HR = 0.51, 95%CI = 0.32–0.80, *p* = 0.003), the PD-L1-low group had better survival than the PD-L1-high group (HR = 0.57, 95%CI = 0.38–0.87, *p* = 0.008), and the RT group had better survival than the non-RT group (HR = 0.48, 95%CI = 0.31–0.73, *p* < 0.001). For the RT group, consistent with the overall group, patients with positive margin status had worse overall survival than patients with negative margin status (HR = 3.57, 95%CI = 1.81–7.01, *p* < 0.001), the RS group had better survival than the RR group (HR = 0.38, 95%CI = 0.21–0.67, *p* = 0.001), the PD-L1-low group had better survival than the PD-L1-high group (HR = 0.44, 95%CI = 0.26–0.74, *p* = 0.002). On the other hand, for the non-RT group, there was no association between the margin status or radiosensitivity or PD-L1 level and the prognosis of patients (*p* > 0.05).

**TABLE 2:** The multivariate analysis of overall, RT, and non-RT groups of patients with locally advanced HNSCC.

Characteristics	Overall HNSCC		RT group		Non-RT group	
	HR (95%CI)	*p*-Value	HR (95%CI)	*p*-Value	HR (95%CI)	*p*-Value
Age						
High	Reference					
Low	0.91 (0.60–1.38)	0.667	1.02 (0.61–1.71)	0.928	0.50 (0.21–1.21)	0.125
Gender						
Female	Reference					
Male	**0.59 (0.38–0.92)**	**0.020**	0.63 (0.35–1.14)	0.127	**0.45 (0.21–0.96)**	**0.039**
Stage						
Stage III	Reference					
Stage IV	1.38 (0.60–3.19)	0.447	1.53 (0.51–4.57)	0.450	4.66 (0.77–28.22)	0.094
T stage						
T1–2	Reference					
T3	1.25 (0.63–2.47)	0.527	1.17 (0.46–2.96)	0.746	2.92 (0.81–10.54)	0.102
T4	0.92 (0.42–2.04)	0.840	0.68 (0.23–1.97)	0.479	1.24 (0.26–5.93)	0.791
N stage						
N0	Reference					
N1	1.05 (0.61–1.79)	0.863	0.62 (0.31–1.25)	0.181	**4.34 (1.43–13.12)**	**0.009**
N2–3	1.19 (0.62–2.28)	0.606	1.07 (0.47–2.44)	0.874	0.86 (0.24–3.05)	0.816
NA	0.00 (0.00–Inf	0.995	0.00 (0.00–Inf	0.996	NA	NA
HPV status						
Negative	Reference					
Positive	0.21 (0.02–2.30)	0.202	0.34 (0.03–4.54)	0.414	0.00 (0.00–Inf	0.997
NA	0.80 (0.30–2.09)	0.642	0.75 (0.24–2.37)	0.626	0.38 (0.03–4.14)	0.425
P16 status						
Negative	Reference					
Positive	0.89 (0.16–5.09)	0.897	0.36 (0.03–4.07)	0.405	8.28 (0.23–302.13)	0.250
NA	1.08 (0.45–2.57)	0.867	1.17 (0.38–3.53)	0.788	1.10 (0.19–6.33)	0.918
Grade						
G1–2	Reference					
G3–4	0.95 (0.60–1.52)	0.845	1.01 (0.59–1.75)	0.960	1.11 (0.37–3.32)	0.857
NA	0.92 (0.11–7.57)	0.940	1.12 (0.13–9.75)	0.922	NA	NA
Margin status						
Negative	Reference					
Close	0.47 (0.18–1.22)	0.119	0.20 (0.03–1.47)	0.113	0.65 (0.19–2.18)	0.481
Positive	**2.73 (1.57–4.75)**	**<0.001**	**3.57 (1.81–7.01)**	**<0.001**	1.00 (0.25–4.04)	0.997
NA	0.80 (0.37–1.74)	0.571	0.91 (0.34–2.41)	0.846	0.55 (0.09–3.21)	0.504
Radiosensitivity						
RR	Reference					
RS	**0.51 (0.32–0.80)**	**0.003**	**0.38 (0.21–0.67)**	**0.001**	0.60 (0.26–1.41)	0.244
PD-L1 level						
High	Reference					
Low	**0.57 (0.38–0.87)**	**0.008**	**0.44 (0.26–0.74)**	**0.002**	0.68 (0.31–1.49)	0.335
Radiotherapy						
No	Reference					
Yes	**0.48 (0.31–0.73)**	**<0.001**	NA	NA	NA	NA

Note. The significant results of multivariate Cox analysis are bolded.

RT, radiotherapy; HNSCC, head and neck squamous cell carcinoma; HR, hazard ratio; RR, radioresistant; RS, radiosensitive; NA, not available.

### Differentially Expressed Gene Identification Between Radioresistant and PD-L1-High and Other Groups

**FIGURE 4:**
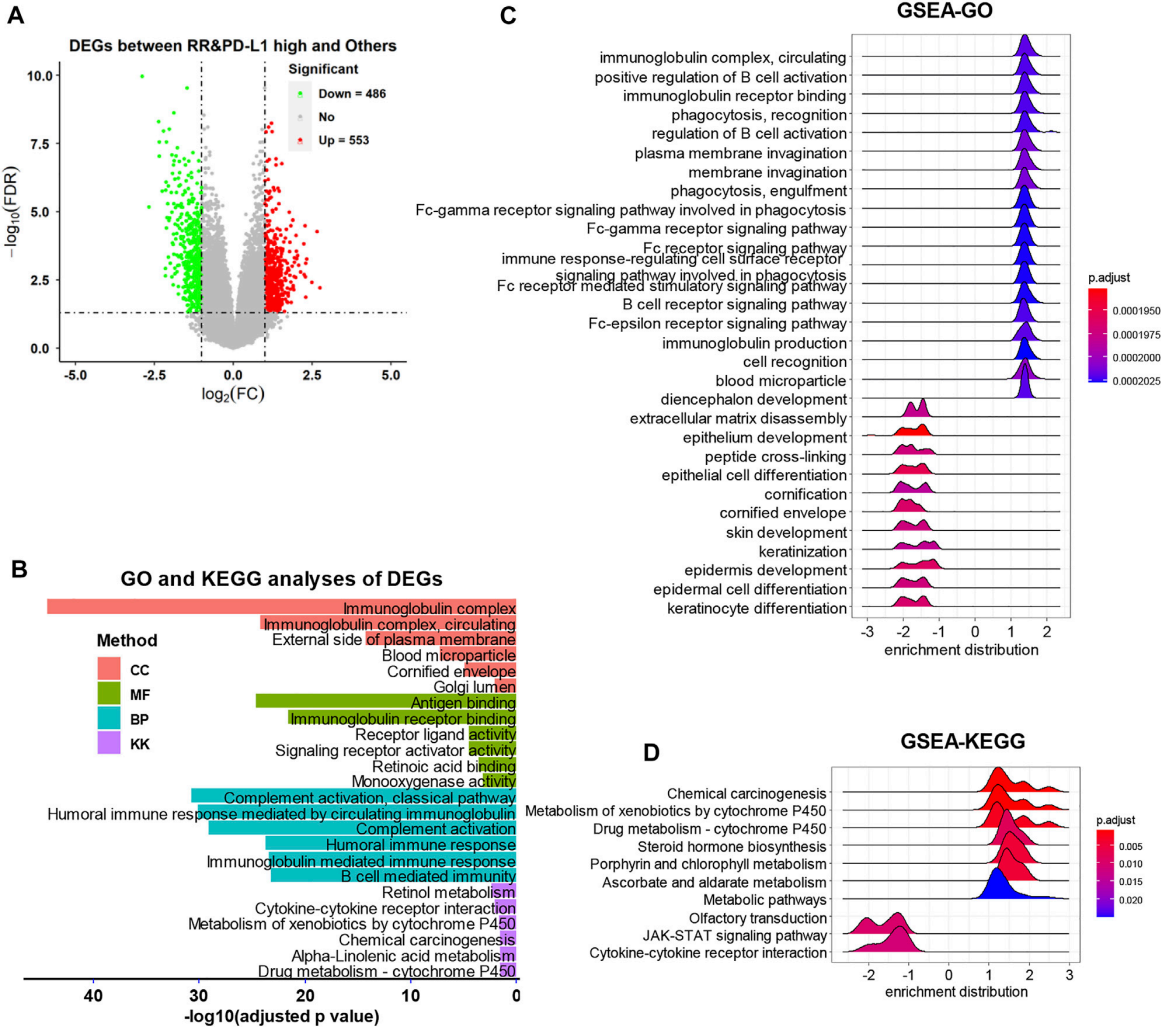
The DEG identification and related functional analyses. **(A)** The volcano plot of DEGs. **(B)** The GO and KEGG analyses of DEGs. **(C and D)** The GSEA of DEGs. DEG, differentially expressed gene; GO, gene ontology; KEGG, Kyoto Encyclopedia of Genes and Genomes; GSEA, Gene Set Enrichment Analysis.

As the volcano plot shows (Figure 4A), the DEG analysis identified 553 upregulated DEGs and 486 downregulated DEGs (Supplementary Table S1, *p*-adjust value <0.05). The functional analyses found that the immunoglobulin, immune response, and metabolism-related items were strongly associated with the DEGs by GO and KEGG analyses (Figure 4B, Supplementary Table S2–5). The GSEA also observed that the immunoglobulin and cell metabolism-related items were upregulated in the RR and PD-L1-high group, while the epithelium development and differentiation-related items and the JAK-STAT signaling pathway were downregulated in the RR and PD-L1-high group (Figures 4C,D, Supplementary Table S6).

### Survival Analysis of Each Differentially Expressed Gene and Hub Survival Gene Selection

The DEGs were further analyzed by univariate Cox model to acquire the survival-related DEGs for locally advanced HNSCC patients who had RT. A total of 213 survival-related DEGs were identified (Supplementary Table S7, *p* < 0.05). Among these survival-related DEGs, there were 123 survival favorable genes and 90 survival unfavorable genes. A univariate Cox model was also performed among locally advanced HNSCC from the GEO database (Supplementary Table S7, *p* < 0.05). The intersect 15 survival-related genes between TCGA and GEO databases were selected for further analysis.

**FIGURE 5:**
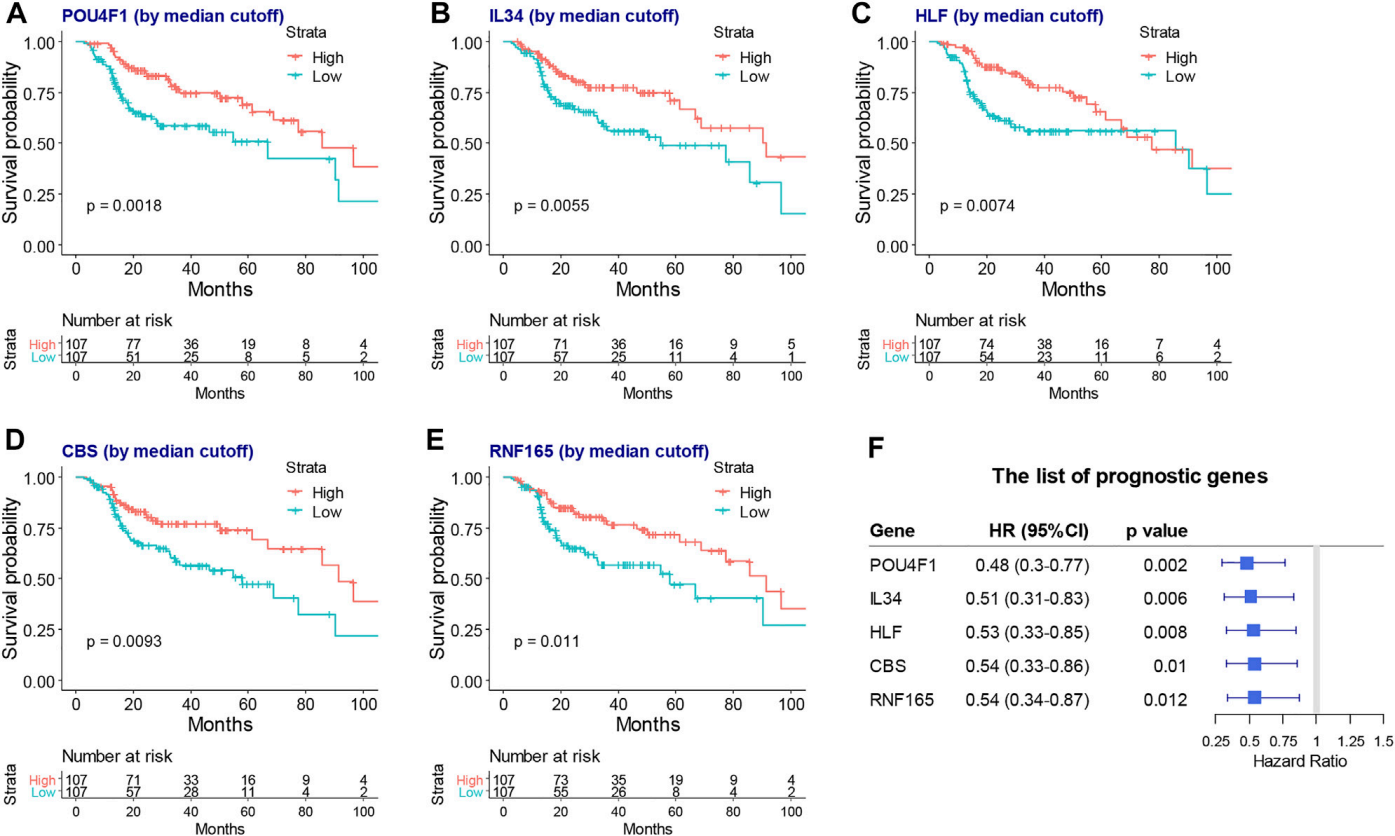
The KM plots and forest plot of lasso selected hub survival genes. The KM plots **(A–G)** and forest plot **(H)** of *POU4F1*, *IL34*, *HLF*, *CBS*, and *RNF165*. KM, Kaplan–Meier; lasso, least absolute shrinkage and selection operator.

Next, a lasso-based Cox model was performed for locally advanced HNSCC patients who had RT by including the intersect survival-related genes. *POU4F1*, *IL34*, *HLF*, *CBS*, and *RNF165* were identified as hub survival genes, as they had nonzero coefficient values by the lasso analysis (Supplementary Table S8). The KM plots and forest plots of these hub survival genes are shown in Figure 5.

### Risk Score Model Construction and Validation

**FIGURE 6:**
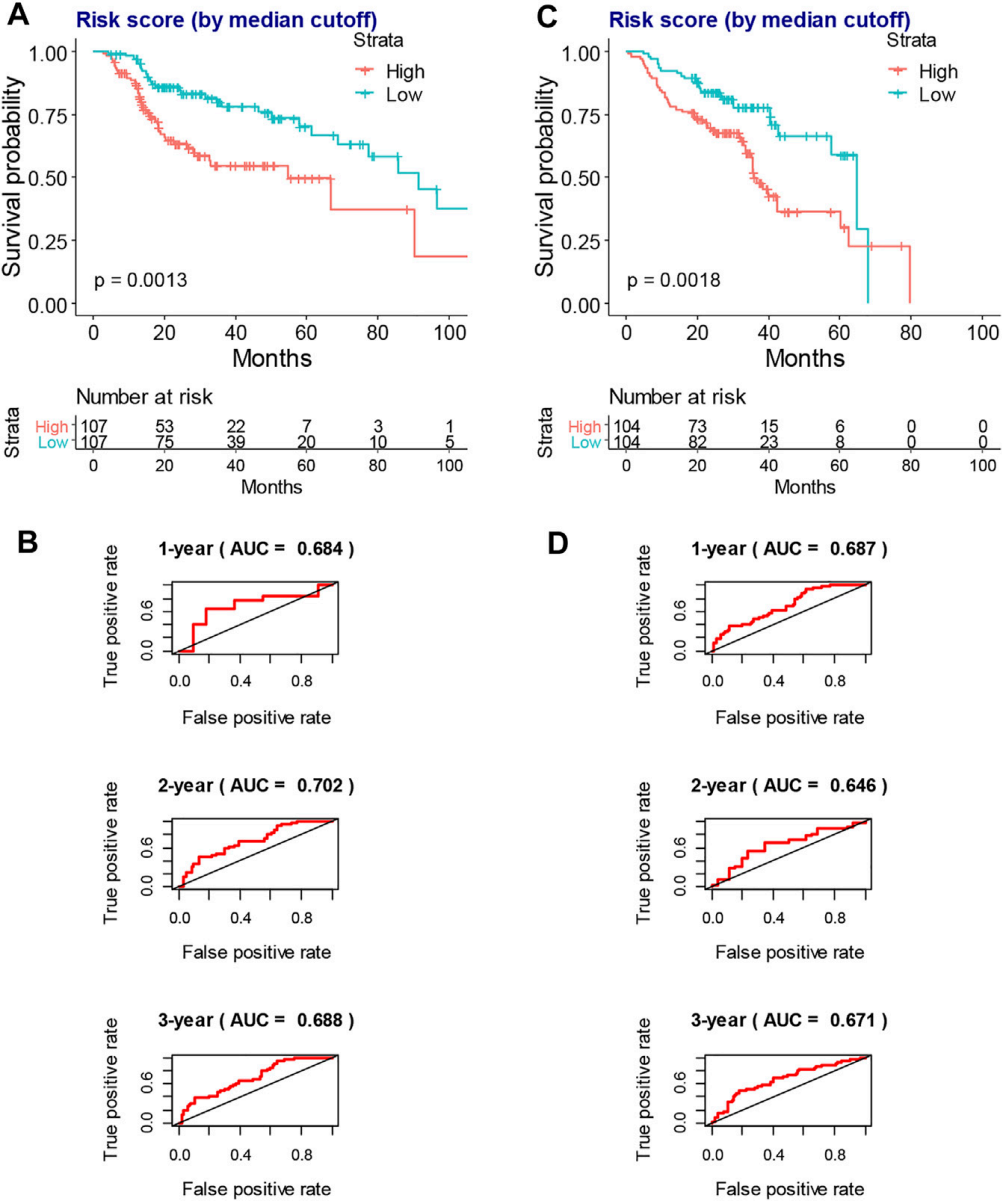
The construction and validation of risk score model in the overall survival of locally advanced HNSCC patients. **(A)** The KM plot shows that higher risk score indicates worse overall survival of locally advanced TCGA-HNSCC patients who had RT. **(B)** The AUC plots of risk score to predict the 1-, 2-, and 3-year overall survival of locally advanced TCGA-HNSCC patients who had RT. **(C)** The KM plot shows that higher risk score indicates worse overall survival of locally advanced GSE65858-HNSCC patients. **(D)** The AUC plots of risk score to predict the 1-, 2-, and 3-year overall survival of locally advanced GSE65858-HNSCC patients. HNSCC, head and neck squamous cell carcinoma; KM, Kaplan–Meier; TCGA, The Cancer Genome Atlas; RT, radiotherapy; AUC, area under the receiver operating characteristic curve.

A risk score model based on hub survival genes was further constructed. The KM method together with the exploration of optimal cutoff found that a higher risk score was significantly associated with the inferior overall survival of locally advanced HNSCC patients who had RT (Figure 6A, *p* = 0.0013). The AUC plots showed that the risk score had a good prediction value (1-year AUC = 0.684, 2-year AUC = 0.702, and 3-year AUC = 0.688, Figure 6B). A validation of this risk score model was performed for locally advanced HNSCC patients from the GEO database (log-rank *p*-value = 0.0018, 1-year AUC = 0.687, 2-year AUC = 0.646, and 3-year AUC = 0.671, Figures 6C,D).

### Estimation of Immune Cell Fractions Between Radioresistant and PD-L1-High and Other Groups

**FIGURE 7:**
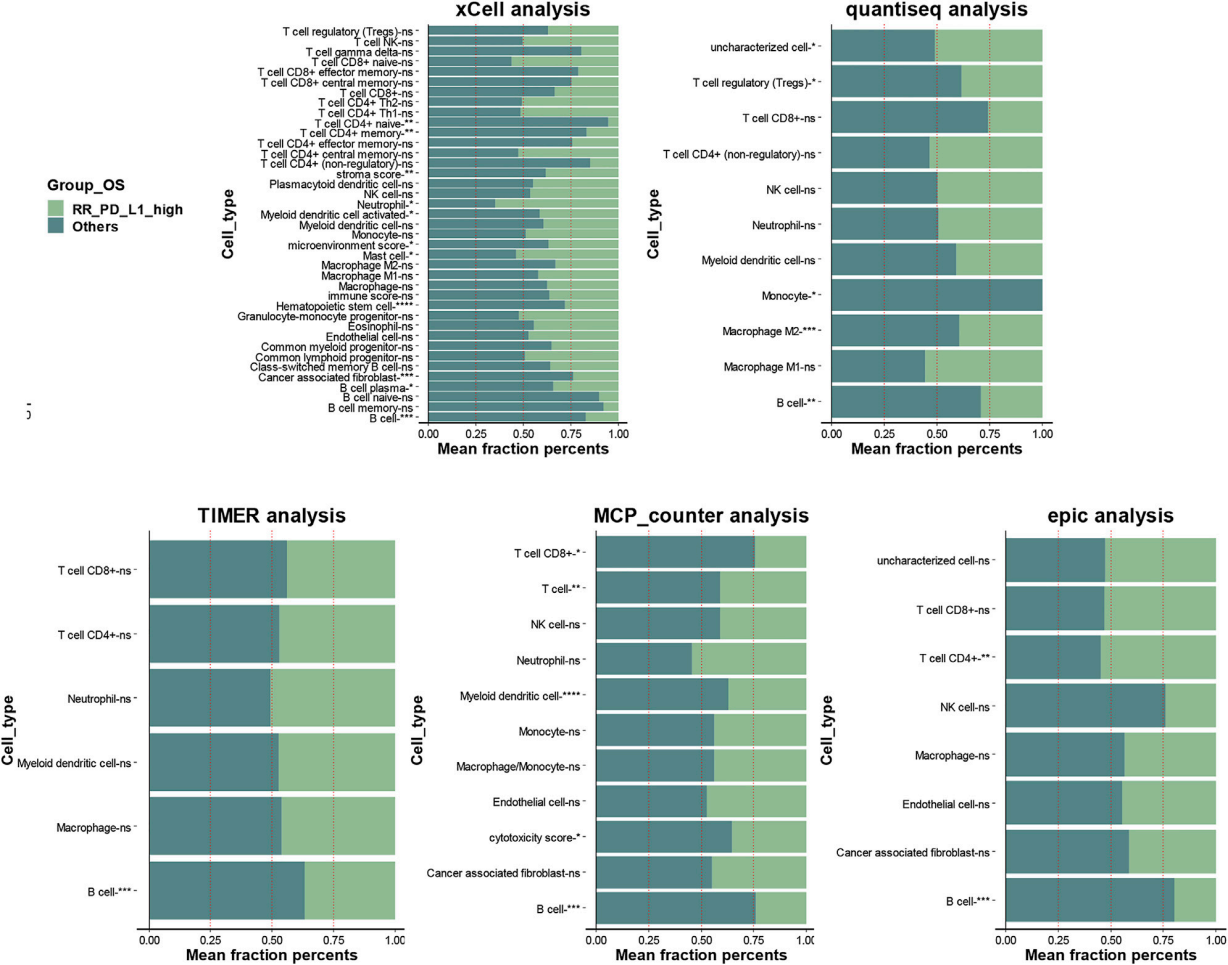
The association between infiltration level of immune cells and the RR and PD-L1-high group in locally advanced HNSCC patients who had RT. The xCell analysis, quantiseq, TIMER, MCP-counter, and epic analyses of the RR and PD-L1-high group and “others” group; the *x*-axis is the mean cell fraction of specific tumor immune contexture. RR, radioresistant; HNSCC, head and neck squamous cell carcinoma.

As shown in Figure 7, xCell analysis, quantiseq, TIMER, MCP-counter, and epic all identified that the RR and PD-L1-high group had lower B-cell infiltration than the others (*p* < 0.05, Figure 7 and Supplementary Table S9–3). The detailed immune cell fractions level calculated by five algorithms are listed in Supplementary Table S9–13.

### Identification of Trichostatin A as the Potential Drug for Radioresistant and PD-L1-High Group

As shown in Table 3, the CMap analysis found that trichostatin A was the potential drug that might reverse the phenotype of the RR and PD-L1-high group (*p* < 0.05 and FDR < 0.1).

**TABLE 3:** Key drugs that had potential therapeutic effects on RR and PD-L1-high group.

Drug	*p*-Value	FDR
Trichostatin A_MCF7	4.55E−17	1.63E−13
Trichostatin A_HL60	1.50E−06	2.68E−03
Trichostatin A_PC3	6.06E−06	7.25E−03

Note. RR, radioresistant; FDR, false discovery rate.

## Discussion

Higher PD-L1 gene expression was found to be associated with an inferior prognosis of head and neck cancer (Lin et al., 2015; García-Pedrero et al., 2017; Müller et al., 2017). The current study first combined the 31-radiosensitivity gene signature and the PD-L1 gene expression to predict the prognosis of locally advanced HNSCC. We found the RR and PD-L1-high group had significantly worse survival than others when HNSCC patients were treated with RT. The RR and PD-L1-high group might potentially benefit from a combination of RT and immunotherapy, which requires further clinical trials.

RS tumors are more frequently present in patients with a phenotype of immune activation (Strom et al., 2017). The estimation of immune cell fractions in HNSCC found that the B cells were significantly higher in other groups than in the RR and PD-L1-high group. It was identified by five different algorithms. A systematic review showed that B cells play a beneficial role in the majority of cancer types (Wouters and Nelson, 2018). A B cell-associated signature was found to distinguish HPV-positive from HPV-negative HNSCC (Wood et al., 2016). High abundance of B cells in the tumor microenvironment and high density of direct B cell/CD8+ T-cell interactions were found to be associated with an excellent prognosis of oropharyngeal squamous cell carcinoma patients (Hladíková et al., 2019). Moreover, B cells could improve the overall survival in HPV-associated HNSCC and be activated by radiation and PD-1 blockade (Kim et al., 2020). We found the B cells were significantly lower in the RR and PD-L1-high group, which proved the new insight into the association between B cells and HNSCC prognosis.

The function analysis revealed several items that were associated with the RR and PD-L1-high group. Among them, many metabolism-related pathways were found to be upregulated in the RR and PD-L1-high group, such as metabolism of xenobiotics by cytochrome P450, drug metabolism–cytochrome P450, ascorbate and aldarate metabolism, and porphyrin and chlorophyll metabolism. Metabolic reprogramming is considered one of the hallmarks of cancer (Hanahan and Weinberg, 2011). Previous studies identified that the changes in glucose, mitochondrial, lactic acid, and other metabolic processes influenced cellular radioresistance (Tang et al., 2018). Our study again proved the importance between metabolism and radioresistance. However, this needs to be further explored by detailed molecular experiments.

By using the lasso analysis, we built a risk score model for TCGA patients with locally advanced HNSCC who had RT. *POU4F1*, *IL34*, *HLF*, *CBS*, and *RNF165* were identified as hub survival genes. We found that higher expression of these five genes was associated with favorable overall survival. The HPV-associated HNSCC has a better survival rate due to a higher sensitivity to chemotherapy and RT as compared with HPV-unrelated HNSCC (Liu et al., 2018a). POU4F1 as a cellular transactivator was shown to be expressed at elevated levels in squamous cell carcinoma of the cervix and to activate the expression of HPV E6 mRNA (Ndisang et al., 2009). It was also found that the POU4F1 suppressed tumor metastasis via c-MET/STAT3 inhibition and EMT suppression in thyroid cancer (Jung et al., 2020). Interleukins could directly or indirectly stimulate cancer-cell proliferation, survival, and diffusion, which are secreted from cancer cells or immune cells among the tumor immune microenvironment. IL-34 plays a controversial role in cancer. In some cancers, IL-34 inhibits cancer cell proliferation and motility, as well as monocyte-like cell differentiation, such as lung cancer (Wang et al., 2021), while it also plays a tumor-promoting role by acting directly on transformed cells to increase their proliferation in a series of cancers (Franzè et al., 2020). Downregulation of the circadian rhythm regulator HLF promotes multiple-organ distant metastases in non-small cell lung cancer through PPAR/NF-κB signaling (Chen et al., 2020). Lower HLF expression was correlated with more advanced renal cell carcinoma (RCC) (Huang et al., 2020) and HNSCC (Liu et al., 2021). Positive CBS is closely associated with a decreased overall survival in patients with gallbladder squamous cell carcinomas or gallbladder adenocarcinomas (Li et al., 2020b). Reduced CBS expression was observed to be significantly correlated with higher tumor stage, higher grade, and shorter overall survival of hepatocellular carcinoma (Kim et al., 2009). No evidence was found between RNF165 and cancers. Few studies were found on these key survival genes and HNSCC. Our study proved new indications between these genes and radiosensitivity, immune response, and the prognosis of HNSCC.

RT was identified to be associated with the activation of the immune system. The radiation induces tumor cell death and the release of cancer neoantigens and damage-associated molecular patterns (DAMPs). The DAMPs would recruit and facilitate the maturation of the antigen-presenting cells (APCs) in their uptake in the irradiated cancer cells. The APCs would then present the cancer neoantigens to T cells. The T cells would be activated and migrate to the tumor microenvironment including primary tumors and non-irradiated tumor metastases, exerting the antitumor effect, which is defined as the abscopal effect (Liu et al., 2018b). However, the binding between the PD-L1 of the tumor cell and the PD-1 of the T cell would inhibit immune responses. The development of an ICI that targets the PD-1 and PD-L1 was shown to enhance the antitumor immunity of RT (Liu et al., 2018b). The combination of RT and anti-PD-1/PD-L1 antibody is a promising strategy supported by a number of preclinical and clinical evidences (Sato et al., 2020).

Utilizing CMap analysis, we found that trichostatin A, which is a pan-histone deacetylase (pan-HDAC) inhibitor of class I and II HDACs, could reverse the phenotype of the RR and PD-L1-high group. Through the deacetylation of histone proteins, HDACs play a critical role in the regulation of transcription (Kulka et al., 2020). The upregulation of specific HDACs has been found in a series of cancers that included HNSCC (Kumar et al., 2015; Li and Seto, 2016). There was a synergistic antitumor activity of HDAC inhibitor SAHA and EGFR inhibitor gefitinib in HNSCC (Citro et al., 2019). The combined use of HDAC inhibitor and RT led to a synergistic effect, which reduced toxicity and diminished intrinsic and acquired resistance in cancer patients (Shirbhate et al., 2020). A study found that trichostatin A increased radiosensitivity of tongue squamous cell carcinoma via miR-375 (Jia et al., 2017). Besides, HDAC inhibitors were found to be associated with immunotherapy. HDAC inhibitors could enhance the expression of cancer antigens, decrease immunosuppressive cell populations such as the myeloid-derived suppressor cells, and induce specific chemokine expression on T cells (Banik et al., 2019). HDAC inhibition was found to potentiate antitumor activity of macrophages and enhance anti-PD-L1-mediated tumor suppression (Li et al., 2021). Our study showed that trichostatin A had the potential to reverse the RR and immune suppression status of HNSCC and might thereby improve the prognosis of HNSCC, providing new bioinformatics evidences on the relationship between HDAC inhibitors and HNSCC. The combination of HDAC inhibitor with immunotherapy and RT might improve the therapeutic efficacy of HNSCC.

Our study had some limitations. First, TCGA is a retrospective database, which has a lower level of evidence. Second, the clinical information from TCGA-HNSCC was often missed due to parameters such as surgery and chemotherapy application, the detailed information of RT, loco-regional control or response to RT, and HPV status. Our heterogeneous cohort in terms of grade or other clinical information might contribute to the different prognoses. Therefore, a prospective study of a homogenous HNSCC cohort is required to validate our study. Third, the GSE65858 from the GEO database had no information of RT experience, so we included all the locally advanced HNSCC samples for the survival analysis. We expect our results to be externally validated by a future study regarding RT and HNSCC. Fourth, the underlying mechanisms of HNSCC and the hub survival-related genes or trichostatin A should be further explored and characterized.

## Conclusion

Our study found that a combination of 31-radiosensitivity gene signature and the expression level of PD-L1 had the potential prognostic value for patients with locally advanced HNSCC who had RT. The RR and PD-L1-high group was significantly associated with worse clinical survival than other patients. The B cells were lower in the RR and PD-L1-high group. The identified risk gene signature of patients with locally advanced HNSCC and the potential therapeutic drug trichostatin A for the RR and PD-L1-high group are worth to be further investigated. Prospective studies are required to validate our findings in a homogenous, prospectively treated patient cohort.

## Data Availability

The original contributions presented in the study are included in the article/Supplementary Material. Further inquiries can be directed to the corresponding author.
